# Formative Evaluation of Suicide Prevention Websites for Men: Qualitative Study with Men at Risk of Suicide and with Potential Gatekeepers

**DOI:** 10.2196/59829

**Published:** 2025-02-26

**Authors:** Doreen Reifegerste, Anna J M Wagner, Lisa Huber, Manuel Fastuca

**Affiliations:** 1 School of Public Health Bielefeld University Bielefeld Germany; 2 Centre for Language Studies Radboud University Nijmegen The Netherlands; 3 GIM Gesellschaft für innovative Marktforschung mbH Heidelberg Germany

**Keywords:** mental health, suicide prevention, men’s health, evaluation, website, gatekeeper, suicide, male, suicide risk, digital communication, intervention, suicidal behavior, digital intervention, digital media

## Abstract

**Background:**

The suicide rate among men exceeds that of women worldwide. One important measure in suicide prevention for men is digital communication interventions, as they enable easy and anonymous access to information resources. This is especially important for men who might not be reached by traditional, in-person prevention methods. Thus, as part of an interdisciplinary project on suicide prevention for men, two specific digital communication prevention measures were developed: (1) a website to inform men at risk about suicide prevention, and (2) a website to educate potential gatekeepers who are in contact with men at risk of suicide about appropriate life-saving measures. Both websites needed evaluation to explore how they are perceived by (1) men and by (2) potential gatekeepers of men at risk of suicide. This is crucial, as existing research lacks formative evaluation that informs the development of intervention communication materials.

**Objective:**

This study aimed to analyze whether these websites were perceived as (1) comprehensible and engaging, (2) authentic and trustworthy, as well as (3) useful by (potential) users. Furthermore, we examined (4) additional ideas for effective communication about suicide prevention.

**Methods:**

We conducted (1) individual videoconference interviews with 24 men to evaluate the website and (2) four focus groups with 8 gatekeepers in each group (32 participants) to evaluate the online education program. The focus group sample was equally distributed regarding gender and age. Recruitment was conducted together with a field research partner who posted adverts on Facebook and Instagram (Meta) to reach as many potential participants as possible in an efficient way. All participants were asked to evaluate the intervention materials using a fictitious scenario of a man experiencing a mental health crisis before the interviews or focus groups took place.

**Results:**

The videos were perceived as (1) catchy, comprehensible, and empathetic, but too long for a short introduction. A balanced mix of emotional and informative content was considered appropriate and helpful. The health information provided was perceived as (2) serious and trustworthy due to citing scientific institutions and video material of men who had experienced suicidal ideation. (3) The intervention’s applicability for men experiencing acute crisis was critiqued, but it was regarded as very useful for comprehensive information. (4) Further communication channels and addressing other male subgroups or gender identities were presented as possible extensions of the program.

**Conclusions:**

Effective suicide prevention research should address both the groups at risk and their support network. Digital communication interventions can provide low-threshold access. Videos with personalized examples are important to give men someone to identify with, which validates their emotional responses and supports their self-esteem, while videos with experts provide relevant and credible information.

## Introduction

Suicide rates among men surpass those among women worldwide. For instance, in Germany, approximately 75% of all suicides are carried out by men [1]. Even though recent reviews have emphasized advances in our understanding of risk factors and countermeasures for suicidal behavior in men and women [2,3], it remains a reality that men are at substantially higher risk. Preventive measures are hence needed that specifically address men and consider the particular needs of men at risk and their suicide-related communication [4].

One important measure in suicide prevention is digital communication interventions. Digital media such as websites are used as information sources on suicide methods but could also be used as easily accessible sources of suicide prevention support [5]. As they enable easy and anonymous access to information resources, digital media can be beneficial tools for a variety of different population groups [6]. Men in particular might profit from digital interventions, as they are less likely to be reached by traditional, in-person prevention methods that might not be tailored to their needs [7]. Recent systematic reviews [8,9] show that communication-based digital interventions appear to be effective measures for male suicide prevention. While the interventions demonstrate high overall effectiveness on suicide-related outcomes (eg, knowledge, attitudes, and cognitions), one of the reviews [9] also found no adverse effects linked to these interventions among men. This means that the risk associated with digital interventions for men at risk seems to be particularly low.

In addition, interventions for so-called “gatekeepers” (people who are in contact with individuals at risk of suicide, eg, family members or friends) play a central role in suicide prevention [10]. They can be considered to fulfill an important bridging function, extending intervention reach to this group of men at risk and being well-placed to initiate difficult conversations [11]. Sharing distressing personal experiences with trusted social contacts can be an important first step in seeking psychological support, for example in the case of clinical depression [12]. Men seem to be less likely to seek professional or informal help and less likely to communicate directly to their closest contacts about suicidal intentions; however, they still show signs of change (eg, further self-isolation, sudden anger, or hopelessness) before the suicide, which makes gatekeeper interventions all the more important [13,14].

Training gatekeepers on how to approach men at risk, and react appropriately in case of disclosure, is of paramount importance. Based on evidence from a systematic review, gatekeepers can be effective in their bridging function for suicide prevention [15], particularly where training programs succeed in sensitizing gatekeepers and building their competencies for engaging with men at risk [16]. Digital training programs for adolescents have been shown to be successful in the development of gatekeepers’ competencies, such as for teachers and peers [17-19].

To date, however, further evaluations for digital interventions for adult men and their (potential) gatekeepers are required [20]. This especially holds true for qualitative research evaluations, such as with interviews and focus groups. While randomized controlled trials (RCTs) and quantitative evaluations are necessary for testing programs’ effectiveness and have also been completed within this project [11], qualitative methods offer the chance to not only test the usability of the developed programs but can also generate insights extending beyond the specific intervention [21].

We therefore conducted two qualitative studies evaluating (1) a website addressing particularly men at risk about suicide prevention, and (2) a web-based program to educate potential gatekeepers about appropriate and critical life-saving measures. The communication strategies selection for the websites was based on the findings of recent systematic reviews, which focused on effective message strategies and communication campaigns for suicide prevention [8,9]. Here, it was recommended to include testimonials and educational materials. The content was also informed by research on gender-specific suicide-related communication [14] as well as the typical communication behavior of men in the days before a suicide [13]. For more details on the structure, components, and materials of both websites [11,22].

The website is specifically aimed at men experiencing acute (suicidal) crises and provides visitors with information about suicidal experiences and behavior, offers of help, and signposts ways to access health provision and mental health support. Using short videos, men at risk talk about their experiences with suicidal crises and how they found their way out of the crisis. In addition, the videos address images of masculinity, stigmatization, shame, and overcoming obstacles to seeking help. Furthermore, experts from various professions within the health and care sectors provide more detailed information. These experts include a male professor and psychiatrist as well as a female professor and psychologist, all of whom primarily conduct research on mental health and suicide.

The web-based program for gatekeepers is aimed at people who are worried about a loved one and consists of four modules. The first module provides information about suicidal experiences and behavior, detailing warning signs. The second module explores communication and conversation strategies. The third module focuses on support services for signposting. The last module addresses the burden on relatives themselves, and their options for self-care, and gives further recommendations for stress-management strategies.

The web-based program (similar to the website) consists of videos featuring men at risk and experts from the health and care sectors. Participants also learn about communication strategies and conversation skills using exemplar audio recordings of people with suicidal experiences and behavior.

There were multiple focuses when evaluating both programs, given their differing objectives and content. On a more superficial level, usability evaluations were key. These have the objective of analyzing whether the digital interface is easy to understand and appealing to potential users [21]. Usability, in general, “is the ability of a product to be understood, learned, used, and attractive to the user” [21], which emphasizes a user-centered approach and focuses on their satisfaction. In health-related contexts, the credibility and authenticity of information is another important determinant of user satisfaction [23]. Because there is an excess of information available online (of varying quality) and because people at risk are very vulnerable, trust is key to their health information-seeking behaviors [24]. Often, expert sources as well as individuals with personal experience can be convincing in this regard.

Finally, the aspect of perceived utility should be considered, because this also influences the intention to keep using the sites and recommendations to others.

Thus, in our evaluations, we aim to analyze whether the website and the web-based gatekeeper program are perceived as (1) comprehensible and engaging, (2) trustworthy, and (3) useful by the two different audiences. Furthermore, we were interested in (4) additional ideas for the communication of suicide prevention beyond the developed digital interventions for men.

## Methods

### Participants

To pursue these 4 research goals, a 2-step study with both men and gatekeepers was conducted.

For the evaluation of the websites for males, 24 videoconference interviews with men in 3 age groups (18 to 30 years old, 31 to 50 years old, 50 years and older; 8 participants per age group) were conducted.

We included all persons who identified as men and were older than 18 years. We did not explicitly search for men with a history of suicidal behavior nor relatives of men with a history of suicidal experience and behavior given ethical considerations. However, based on the recruitment information (which disclosed the aim of the website as one for suicide prevention), many participants reported that they themselves were affected by suicidal ideation and behavior, or had vicarious experiences through friends, acquaintances, or relatives. We did not exclude these participants from the sample.

For the evaluation of the gatekeeper program, four videoconference focus groups with gatekeepers with 8 people each (32 participants in total) were conducted. The focus group sample was equally distributed regarding gender and age (50%, 16/32 female and 50%, 16/32 male; 50%, 16/32 individuals from younger, ie, under 40 years old, and 50%, 16/32 from older age groups, ie, over 40 years old).

For an overview of the specific age and gender of each participant refer to Table 1. A videoconference format for the interviews and the focus groups was chosen to maximize ease of attendance and the potential for anonymous participation.

**Table 1:** Age and gender of participants in interviews (24 men) and focus groups (16 men and 16 women in 4 groups) for the evaluation of websites for suicide prevention.

Method and gender		Age (years)	Interviewer or moderator	
**Interviews**				
	Male	48	Female	
	Male	63	Female	
	Male	39	Female	
	Male	24	Male	
	Male	48	Male	
	Male	67	Female	
	Male	56	Female	
	Male	66	Female	
	Male	62	Female	
	Male	49	Female	
	Male	30	Female	
	Male	33	Female	
	Male	30	Female	
	Male	29	Female	
	Male	35	Male	
	Male	36	Male	
	Male	40	Male	
	Male	65	Male	
	Male	25	Male	
	Male	24	Male	
	Male	54	Female	
	Male	51	Female	
	Male	20	Female	
	Male	21	Female	
**Focus group 1**				Male, Female
	Female	60		
	Female	58		
	Female	55		
	Female	47		
	Female	41		
	Female	72		
	Female	41		
	Female	51		
**Focus group 2**				Male, Female
	Male	35		
	Male	18		
	Male	36		
	Male	27		
	Male	33		
	Male	34		
	Male	27		
	Male	40		
**Focus group 3**				Male, Female
	Female	22		
	Female	34		
	Female	32		
	Female	24		
	Female	29		
	Female	30		
	Female	37		
	Female	30		
**Focus group 4**				Male, Female
	Male	71		
	Male	44		
	Male	65		
	Male	58		
	Male	61		
	Male	75		
	Male	46		
	Male	41		

Sample sizes were therefore consistent with recommendations for sample sizes in interviews and focus groups to reach sufficient saturation [25]. As it happens, data saturation in the interviews was already reached after about 20 interviews. The number of people refusing to take part in the study or not showing up at the scheduled time was low to nonexistent (especially in comparison to market research projects).

### Recruitment

Recruitment was conducted together with the field research partner Quotapoint Pharma and Health in Germany, which specializes in recruiting participants for qualitative studies on health-related topics. They used an announcement in their newsletter (refer to Multimedia Appendix 1 for an English version of the recruitment material). They also posted adverts on Facebook (to reach potential participants older than 35 years) as well as Instagram (to reach potential participants younger than 35 years). This approach to recruitment was chosen in order to reach as many potential participants as possible in an efficient way. After the initial contact was made, a screening process was carried out using a screening tool, with which suitable participants were filtered according to the sample criteria. During the screening process, a brief sociodemographic check was conducted due to the all-male target group for the interviews and the gender-mixed sample for the focus groups. Following this, a trigger warning was given that the subsequent screening interview would be about suicide prevention to minimize the psychological stress on the potential participants. Participant recruitment was documented with regard to the filtering criteria, for example, age or gender, and checked before the participants were finally invited to the interviews and focus groups.

### Ethical Considerations

Ethical approval for this study was obtained from the Ethics Committee of Bielefeld University (number 2023-153). All participants were provided with an information form describing the study (refer to Multimedia Appendix 2 for an English translation of this consent form). Participants in both studies were required to return signed consent before they could participate in the study, via a web-based form and a data protection agreement. As the interviews and focus groups took place as a videoconference, the participants were once again reminded about their data and confidentiality rights at the start of the interviews. The interviews were recorded only once active consent to recording had been given by the participants.

As stated above, we avoided explicitly searching for men with a history of suicidal behavior consideration due to ethical considerations.

All data were collected anonymously. All participants were provided with financial compensation in recognition of time spent and their contribution to the study (€90 [US $94] per participant in interviews; €140 [US $146] per participant in focus groups).

### Procedure

After giving their consent, all participants were asked to extensively study the intervention materials and briefly evaluate them via a quantitative survey. This was both for the website for men at risk and the gatekeeper online program (step 1), and was completed before the interviews and focus groups took place (step 2). In the first step, called the pretask, the participants were asked to engage with and reflect on the content in a home setting. Initial findings on the attractiveness, intuitiveness, and comprehensibility of the content were collected using short questions via online survey software. Surveys took approximately 5-10 minutes to complete. Responses from the surveys were included as context information in the qualitative data analysis and were used during the interviews as conversation starters.

In the second step, the interviews and focus groups were conducted in July 2023 using the ZoomX videoconferencing platform (Zoom and Telekom), which is hosted in Germany. After a brief summary of the project (and in the case of the focus group a round of introduction), participants were informed about the research goals, their ability to end participation at every step of the data collection, and about the applicable data protection rules. Moreover, they were given the opportunity to ask questions before the interviews and focus groups began.

All interviews were conducted by one of two authors (LH and MF; one male and one female) and ran for 45 minutes. All focus groups were conducted jointly by one moderator and one assistant and ran for approximately 90 minutes (refer to Table 1 for details on role allocation). These were recorded, using both audio and video. During the interviews, the interviewer took notes on the content of participant discussions. Meanwhile, during the focus groups, the assistant listened to the discussion of the moderator and participants, while taking handwritten notes on all discussion points.

### Materials

The website for men with an increased risk of suicide entitled “Männer-stärken” (empowering or strengthening men) offers information on suicide prevention. The website contains videos, information summaries, and lists of helpful recommendations. Complex content is conveyed in simple language and with the help of visual illustrations. Long text sections are avoided in favor of concise information summaries and lists. Graphic illustrations feature a line art and watercolor design. The website is primarily designed in yellow-themed color.

The web-based program “Help for Relatives” provides information on the topic of suicide or suicide prevention in men, with the help of text content on the website and an accompanying manual, as well as four videos approximately 20 minutes long each. It is structured into four modules: (1) suicidal ideation and behavior, (2) communication, (3) offers of assistance, and (4) self-care. Here, visual elements are additionally used to convey complex information.

The short, standardized web-based surveys conducted next assessed a range of topics relevant for website evaluation (confirming participant identity, first impressions of the web-based platform, liking or disliking different aspects as open text fields and grading items as well as optimization potential) comprising closed- and open-ended questions. Their results were used as conversation prompts to provide an easy entrance into the interview situation and generate conversation.

The interviews and focus groups were structured, with a series of guiding questions and prompts exploring the views of men and gatekeepers on the potential design, content, functioning, and user experience (eg, optional phone and email support) of a web-based navigation tool (eg, liking or disliking the online platform for different aspects such as understandability, authenticity, the trustworthiness of given information, and the usability in everyday life and in acute crisis). Questions and prompts were developed based on mental health research as well as usability research. Participants were encouraged to share their perspectives verbally with the group or the interviewer.

Several techniques were used to deal as sensitively as possible with the topic of suicide. One technique was the scenario technique or vignette methodology [26], which was used to keep the interviewees’ psychological stress as low as possible and enable them to evaluate the digital interventions on the basis of a standardized crisis situation: a fictitious scenario of a man in a psychological crisis was presented, which served as the basis for the interviews. In addition (and also to minimize emotional stress), we first asked the men indirectly about the assumed perceptions of others, before they were asked to present their own views. This so-called circular questioning is a technique often used in therapy to investigate sensitive topics [27]. All materials were pretested in advance with persons matching the sample structure (refer to Multimedia Appendix 3 for the guides and short protocols of the interviews and the focus groups).

### Data Analysis

Regarding personal reflections that were relevant to our analysis, there were methodological as well as gender aspects to consider during the data analysis phase. One part of the team comprises established scientific researchers, who are rooted in theory-based quantitative and experimental as well as qualitative research, while the other part has long experience in market research for new websites and consumer products. We tried to link these 2 worlds and systemize the content of the interviews and focus groups against the background of theories like the unified theory of acceptance and use of technology and similar theories [28] as well as known consumer needs for products in digital environments (Multimedia Appendices 4 and 5). Such products, like cars or digital devices also often address a primarily male audience.

At the same time the three researchers in the team who identify as female tried to be especially empathetic to male perspectives, whereby we for instance explicitly asked for input from the male researcher in our team.

Taking into account the reflexivity of the researchers, it is important to reflect on the different perspectives and life experiences of the researchers who did the coding (a man over 50 and a woman under 30) in terms of how this could influence the analysis of the data. In the sense of Clark and Braun [29], this serves to make the subjective influences of the researchers transparent and to consider them as a research resource rather than a bias. On the one hand, it is therefore important to emphasize age and generational perspectives. The male researcher over the age of 50 years has a more experienced perspective than the female researcher under the age of 30 years, which proved to be a suitable age structure with regard to the sample of interviewees aged 18-60 years, as all age splits could be covered within the researcher perspectives. At the same time, generationally different views on suicide, suicide prevention, and mental health can be countered from an age perspective. Furthermore, by choosing a man and a woman to analyze and collect the data, gender-specific needs of the participants and insights can be gained from the data. As a man, the male researcher is more attuned to the lifeworld issues and needs of the male target group, whereas the female researcher is more sensitive to gender-specific differences. Finally, the degree of digitality can also be reflected in the analysis and collection of data. It can be assumed here that the younger female researcher has a high level of familiarity with digital technologies and communication styles, which could make a difference in the consideration and interpretation of what the participants say with regard to the user-friendliness and address of the websites. Due to the selected age range of the sample, it is also possible to take into account any degree of digitality of the participants.

Qualitative data were managed using transcripts, an Excel table, and protocols of the interviews and the focus groups. A thematic analysis [29,30] was conducted by 2 authors on interview and focus group data notes, chat conversations, and survey responses to identify and summarize the key topics and preferences raised by the interview and focus group participants within each area of the research questions while preserving the breadth and diversity of perspectives presented. Analyses and developing categories were regularly discussed with all members of the research team.

A combination of deductive and inductive approaches to coding was hence applied to the research. Deductive is here defined as linking the answers to the theoretical and empirical background. More specifically, deductive themes were derived from literature and applied to the material. In contrast, inductive approaches entail being open for topic-specific feedback and including themes that arise from the material and which had not been previously assumed by the researchers.

With regard to the methodological explanations by Braun and Clarke [29] regarding established coding practices, the decision was therefore made to use “mashups,” that is, a mixture of existing literature-based codes and immanent interpretation. This is due to the special feature of the study that, in addition to the content-related exploration of the relevance of such websites for the target group, aspects of user experience were also examined, which sometimes include merely technical aspects.

Before starting the data analysis, an initial broad coding framework was developed based on the key areas of comprehensibility, appeal, credibility, utility, and ideas for further communication measures in relation to the presentation of the website content. The data was coded deductively within each category. As the coding progressed, subcategories that were not included in the deductive framework were developed inductively, such as the relevance of suicide prevention services as a website per se.

## Results

### Comprehensibility and Appeal

With regard to our first aim of analyzing the comprehensibility and appeal of the videos, findings showed that they were perceived as catchy, understandable, and empathetic by both men and gatekeepers, but too lengthy. Men of all 3 age groups evaluated the website as looking serious, clear, and professional. They especially noted the yellow colors as being positive, friendly, and informative. In contrast, they would perceive the color black as being too masculine, sad, and aggressive.

The content was perceived as easy to receive due to simple and understandable language, and plain typography in combination with images and videos that make complex topics accessible to the general public. The combination of images, video, and text was considered appropriate and varied. Especially the graphic illustrations, short sentences, and comprehensible statistics were perceived as more effective than “text deserts” and allow easy understanding:

Numbers also make the whole thing a bit more tangible, I'm perhaps more of a numbers person, so it makes sense in that direction, but I always find it much easier to understand, otherwise you're in kind of an empty space where you talk around [the topic] without having any numbers.

male, 24 years, interview

For some videos in the gatekeeper program, the language from featured experts was perceived as being too scientific that is, using too many scientific terms.

Highly modern or fancy websites were not expected by most viewers given the seriousness of the topic; rather, they expected timeless design and a clear presentation of facts.

If I make the thing colorful and pretty, then it loses a bit of its meaning;

male, >41 years, focus groups

It just has to be good and clear and, not like a thousand other sites, I always knew how to navigate [the site] and found guidance.

male, 62 years, interview

However, participants also rated the website as too unmodern due to long texts and videos. Especially the videos (on average 7-8 minutes and up to 20 minutes in length) were perceived as too lengthy. Feedback indicated that these demanded too much attention and increased the risk of participants discontinuing the program. The same applies to searching and scrolling on the website, which was also perceived as not user-friendly. Respondents suggested making the aim of the website for men at risk and the gatekeepers’ program clearer at first glance. Specifically, it was suggested to offer specific content sections and “trailer-length” videos for a first impression. The option of more detailed formats should be an option for cases where more information is required. Some even wanted more facts and information in this regard.

Some of the men perceived the title of the website for men (which means empowering or strengthening men) as nonintuitive and misleading because they had associations with potency substances or coaching about masculinity.

If I didn't know the context, then I would think of, I'll say, potency or something like that.

male, 66 years, interview

well, strengthening can be in many ways, so that could also be some sexual enhancers or some power food supplement stores for bodybuilders.

male, 51 years, interview

In addition, they found the claim of “strengthening men” rather counterproductive for the destigmatization of mental distress, which they felt should not be connected to weakness.

For me, it's more of an illness that can be treated like any other illness and has nothing to do with strength or weakness. So, I would tend to think that it's the supposedly strong men, who are strong in the classic sense, who can get it because they're not so good at dealing with problems.

male, 67 years, interview

### Credibility and Authenticity

In terms of authenticity and trustworthiness, the information on the website and in the web-based program was perceived as serious and trustworthy due to referencing universities, health insurance companies, and scientific institutions as sources as well as facts and figures. In addition, the perceived clarity, color choice, and timelessness were attributed as professional and serious.

What naturally contributes to credibility is that the logos of the participating universities were right at the front of the home page. Then there were these experts, which of course also increased credibility to a certain extent.

male, 33 years, interview

I found the interview with the professor very, very good, especially because you can see how extensive the problem is, where he had his problems, and to understand that there is a big process behind it.

male, <40 years, focus group

The same perception applies to facts and statistics as an integral part of evidence-based information.

Several men in the interviews stated that it is good that the programs’ developers are based in universities. This lent credibility to the websites, which were perceived as trustworthy. The men were sure that the websites were based on the latest research rather than being driven by advertising or a pharmaceutical company for reasons of profit, even when they did not recognize all the universities involved.

In addition, including links to other sources increased the perception of content presented as authoritative.

Participants also rated the videos featuring men at risk of suicidal behavior as authentic and easy to understand. Including these videos offered visitors faces to identify with, and validation for experiences. It also created the feeling for visitors who self-identified as at risk that they are not alone with problems (even without having spoken to others personally about the topic), making it possible to still feel a sense of belonging.

Especially with the videos, it brings a personal closeness and makes it easier to accept support. Because you realize that there are also real men who have the same problems.

male, 36 years, interview

At the same time, using and engaging with these vignettes can offer a supported form of self-diagnosis which helps men at risk to classify their own feelings and symptoms.

That you can draw parallels from yourself to a description or identify with a person who also describes [these experiences].

male, 56 years, interview

That the men at risk featured in the videos were of various ages was also perceived as helpful to recognize that this is a problem for all ages.

A video featuring individuals sharing their own experiences can also serve a more inspiring purpose. Sharing these experiences and also how that person also managed to recover can demonstrate how this is possible, changing perceptions of how good the chances are of a recovering person also depicts an example of how good the chances of recovery can be:

There were also patients in it, I think several times, I think the same person was in several videos. (Which then takes you by the hand, along the lines of) Look, I'm already here, why don't you come too, you can see I'm not doing so badly. Or it makes me feel better.

male, 30 years, interview

In contrast, the men at risk found archive photos of emotional states on the website for the gatekeepers as noncredible, perceiving these instead as too dramatic and inauthentic. This underlines how important the credibility of the material is, as where material otherwise seems staged, inauthentic, and prompts an adverse reaction:

So, when I'm told what I'm supposed to feel in such a way, I am out… and that's what these stock photos do, because in my opinion they want to create something very specific and emphasize what I as a consumer am supposed to think.

male, <40 years, focus group

### Utility

In terms of utility, the websites are perceived as helpful overall. The participants think that the websites are a good place to find information, especially because men at risk also shared their experiences. Because of this inclusion, the information shared was perceived to be helpful as realistic and practical advice by users. Behavioral recommendations were considered to be helpful tips, which were highly relevant to everyday life.

With regards to the website, one man (21 years) reported in an interview:

I particularly liked the “Glossary of concerns” and the “Addressing distress” pages, because these are the most practical in my opinion. I also really liked the fact that all the texts always included a link to further help or resources. These resources were very well structured based on different needs. Contact details were also given everywhere.

I mean, you get a lot of information so that you can perhaps recognize the signs [...] when you see the symptoms... irritability, listlessness, and so on.

male, 36 years, interview

The gatekeepers reported similar reactions to their program:

It was very much about.... how do I try to understand a person and how do I communicate with that person so that the person appreciates it, that's what you're allowed to do?

male, <40 years, focus group

The participants found digital anonymity on the internet and the possibility of searching for information without being obliged to do something helpful. According to them, it provides men at risk and concerned family members support with low barriers to access because help is provided in anonymous internet spaces for this very sensitive and stigmatized topic. Several men in the interviews and focus groups stated that they experienced less shame when talking in a videoconference about mental health problems and more potential to engage with the idea of getting help, because of the anonymity.

The help site for relatives was considered to provide significant added value emotionally, as it can support the family members to minimize difficult feelings related to powerlessness.

If the people notice something in their surroundings that they can then check with this program as a first step, ok, are these worries that I'm having justified, is this really going in the direction that I'm thinking?

female, <40 years, focus group

At the same time, however, the applicability in crises is questioned as to whether men at risk of suicide in an acute crisis would still visit websites to get help because they might not have the energy to do so:

So within this situation, he might not want to read something for hours or anything like that, but he's overstimulated, so he doesn't know what to do next, he just needs someone with whom he can somehow communicate verbally, to one.

male, 66 years, interview

Some were even concerned that the “unregulated internet space” could promote a problematic approach to the topic of suicide risk and counteract the actual aim of the service. They were worried that such a website could increase the risk of seeking out further information and then receiving “bad advice” or “getting the wrong person” in other online spaces, which would then hinder them from seeking help in “real life.”

Participants suggested prefiltering the offers of help for those at risk in an acute crisis, by providing emergency numbers and direct contact persons or a helpline who can offer help in a crisis. Given that websites provide information asynchronously, the hope for immediate, quick help for relatives in emotional distress (contact details, chats, and direct contact options for immediate personal offers of help) remains unfulfilled.

Table 2 summarizes the answers to RQ1 to RQ3.

**Table 2:** Summary of Results of the Interviews and the Focus Groups and their Feedback on Comprehensibility, Credibility, and Utility of the Websites for Suicide Prevention.

Theme	Interviews with men (24 participants)	Focus groups with Gatekeepers (32 participants)
Comprehensibility and appeal	Videos were perceived as catchy, understandable, and empathetic But too lengthy and too fixed Website is perceived as authoritative, easily understandable Yellow color as positive, friendly, and informative The website title was discussed as it seemed ambiguous	Videos were perceived as catchy, understandable, and empathetic But too lengthy and too fixed Too many scientific terms in the expert videos A modern or fancy design is not expected
Credibility and authenticity	Websites are perceived as trustworthy due to references to scientific institutions as well as facts and figures Generic stock photos provoke adverse responses	Websites are perceived as trustworthy due to references to scientific institutions as well as facts and figures Generic stock photos provoke adverse responses
Utility	A good place to find information But might not be appropriate or helpful in emergency cases	A good place to find information But might not be appropriate or helpful in emergency cases

### Ideas Beyond the Intervention Material: Other Communication Channels

Both the topic, the desire for education about the topic, and its destigmatization were evaluated as critically important, especially from the group of men younger than 30 years. As a consequence, the respondents also recommended using more communication channels for promotion in both the health and public sector to make the information more accessible to everyone. They suggested using all conceivable communication channels to draw attention to this offer of help, to destigmatize the topic, and to widen the reach for education purposes.

I think this should be widely distributed now, because ultimately, of course, I have to reach the men everywhere

man, 51 years, interview

This could involve promoting the websites more prominently on social media channels like Instagram and TikTok (ByteDance), in magazines, outdoor media, TV health shows, cinema and radio spots, at train stations, and in sports stadiums.

They also suggested connecting the website for men at risk more visibly with the online program for gatekeepers. This would mean that where the website for men is mentioned in a private conversation with a friend (gatekeeper) who has already screened for information and helpful content from the website. Conversely, a man in acute crisis could recommend the program for the gatekeepers to his relatives.

While it was considered valuable that topics around masculinity and suicidal ideation are being addressed, suicide prevention services specifically for men were questioned as a concept by the participants. Despite the information about the statistically higher suicide rate among men, some of the content produced on the sites was considered too conservative for the way that gender roles were presented, because they offered no identification potential for other gender identities, such as nonbinary, intersex, or transgender persons.

## Discussion

### Main Results

The goals of the study were to collect information on the comprehensibility, attractiveness, authenticity, and usability of websites for suicide prevention for men in crisis as well as their gatekeepers. Both websites present psychoeducational resources from experts and patients for both this (vulnerable) group at risk and for their support network. Overall, the findings of this study provide valuable insights into the design and usability of suicide prevention websites addressing men in crisis and their gatekeepers and may provide important implications for future digital prevention interventions.

Potential users of the websites evaluated the balanced mix of emotional content by persons at risk and informative content by scientists as adequate, credible, and helpful. This highlights the potential of this particular combination to address stigmatized topics such as suicidality and mental health and aligns with previous research suggesting that personal narratives combined with information may enhance mental health outcomes and decrease stigmatizing attitudes [31]. However, there were also challenges noted by interview participants, who indicated that they felt overwhelmed with video lengths and the multitude of options to navigate on the websites. Studies on video length have shown that videos with a length of more than 6 minutes can reduce interaction and understanding of learning significantly, thereby acting as a barrier to usage [32]. Based on cognitive load theory and taking into account the limited capacity of active working memory, they recommend using short videos that segment information into small blocks [33]. In addition, based on theories of cognitive capacity, studies on website design show how simplified navigation reduces barriers and prolongs usage [34].

Another key area identified was the need for additional communication channels to signpost to and promote the websites. Users expressed the importance of outreach to raise awareness about the websites and options for individualized and interpersonal communication after using the websites, especially in crisis situations. This aligns with research advocating for multiple communication channels in mental health interventions [35].

In sum, our study shows that both websites are perceived as trustworthy and reliable sources for male suicide prevention. Based on the perceived shortcomings of the programs and participants’ recommendations, the study also provides important implications and improvement strategies for future interventions. This includes communicating brief and concise information as well as integrating options for more immediate emergency support. Overall, our findings underline the need for comprehensible, well-structured digital information which provides a balance between information richness and clarity. For both gatekeepers and the men at risk themselves, practical advice should be incorporated into communication interventions, and shorter content formats should be preferred, particularly considering video length. While digital interventions are undoubtedly crucial, our study also reveals the need to embed them in offline contexts and accompany them using other communication channels.

### Limitations

Some limitations of the studies must be noted.

Due to the recruitment strategy used, we had a sample of very open, highly involved, and topic-aware participants who occasionally saw themselves as ambassadors and rated the social relevance of suicide prevention as very important. This means that emphasis on education and destigmatization related to this topic was already important for them. Thus, our results cannot be held to be transferable to people who may have internal barriers and fears about engaging with the topic. Furthermore, our cross-sectional studies focused on short-term effects and website evaluations. A longitudinal study would be needed to evaluate whether the websites are a helpful tool in the long run [36].

With regard to male gender and identification itself, we made broad generalizations, as it was our goal to create information platforms that were as universal and accessible as possible by as many men as possible. However, this also meant that specific challenges and burdens of people identifying as male and related subgroups (such as homosexual men, transgender men, and men from minority ethnic groups) were not specifically taken into account. Here it is pertinent that members of the LGBTQIA+ (lesbian, gay, bisexual, transgender, queer/questioning, intersex, asexual, and others) community are much more prone to suicidal thoughts and mental health problems than the population at large [37]. Developing prevention campaigns and information tools specifically tailored for them is thus of paramount importance. The same applies to other minorities, such as immigrants, non-traditional German-born individuals, older individuals, living alone, or those with wider mental health issues. Future research is needed that examines interventions for those vulnerable groups more closely and works with more diverse samples and communities.

Finally, our studies did not focus on creating an intervention directed at changing masculine tropes. This could be worthwhile, as studies suggest that social media campaigns can trigger conversations about masculinity [38,39], potentially questioning behavior driven by traditional masculine norms and value sets that aggravate the risk of (mental health) distress and negative experiences.

### Conclusion

Both an RCT of the gatekeeper website [11] as well as this qualitative evaluation indicated that the program was positively evaluated by the participants, in the sense that it was seen as comprehensible and informative. The RCT [11] reported that the training of gatekeepers through a relatively short digital training program is effective based on the knowledge gained. In addition, the qualitative research demonstrates that both types of videos included in the program seem to be relevant for knowledge gain. While the videos featuring real individuals constitute important figures for personal identification and validation, supporting emotional health and self-esteem, the expert videos provide relevant and credible information. Such websites can, therefore, support gatekeepers in suicide prevention. However, it should not be overlooked that such interventions could be perceived to function as “tasking” the gatekeeper to manage the situation, thereby placing a specific burden on gatekeepers, who might feel even more responsible for the suicidal ideation and behavior.

Future studies in this area should also develop guidelines for population-specific communication with men at risk as well as gatekeepers.

Findings from brainstorming ideas beyond the tested interventions highlighted the need to integrate such low-barrier digital offers as one step in a strategy for more comprehensive gatekeeper support. Websites for men and gatekeepers can only fulfill one role in this strategy, with regard to national suicide prevention measures, and must be supplemented by other measures, such as a regional care network for mental health, architectural measures at high-risk sites such as overpasses, and helplines provision [4].

## Appendix

Multimedia Appendix 1 Recruitment material.

Multimedia Appendix 2 Consent form.

Multimedia Appendix 3 Protocols for interviews and focus groups.

Multimedia Appendix 4 Guide focus groups.

Multimedia Appendix 5 Guide interviews.

## Abbreviations

LGBTQIA+: lesbian, gay, bisexual, transgender, queer/questioning, intersex, asexual, and others
RCT: randomized controlled trial

## Data Availability

The datasets generated or analyzed during this study, that is, the transcripts of interviews and focus groups are not publicly available due to data protection reasons but specific portions of the data that are sufficiently anonymized may be obtained from the corresponding author on reasonable request.
