# Supplementary material for: Formative Evaluation of Suicide Prevention Websites for Men: Qualitative Study with Men at Risk of Suicide and with Potential Gatekeepers
Source: JMIR Form Res. 2025 Feb 26;9:e59829. doi: 10.2196/59829 (PMC11904374; doi:10.2196/59829)
Supplement: Multimedia Appendix 1 [file formative_v9i1e59829_app1.pdf]

GIM | RELEVANCE COUNTS.

Recruitment material

Suicide prevention

Evaluation website/e-learning tool with information for men with suicidal experiences and behaviour

Screening

Hello, my name is [name] from [agency], we are currently conducting a market research study for a website test. Are you interested in taking part?

Q1. Gender (do not ask)

Female	( )	*[Continue with Q3]*
Male	( )	*[Continue with Q2]*

- ✓ *IDIs: 100% men*
- ✓ *Focus groups: 50% of the sample Women: 100% women in GD2 (younger) & GD4 (older)*

Q2. Men IDIS & FGDs: Please tell me how old you are: (please note and categorise)

___________ years

Under 18 years	( )	*Please exit*
18-40 years	( )	*[Continue with Q4, GD]*
18-30 years	( )	*[Continue with Q4, IDI]*
31-50 years	( )	*[Continue with Q4, IDI]*
41-75 years	( )	*[Continue with Q4, GD]*
51-75 years	( )	*[Continue with Q4, IDI]*
Over 75 years	( )	*Please end*

- ✓ *Quota for men possible for IDIs as well as for groups, can still be handled flexibly here.*

Q3. Women FGDs: Please tell me how old you are: (please note and categorise)

___________ years

Under 18 years	( )	*Please exit*
18-39 years	( )	*[Continue with Q4, GD]*

40-75 years ( ) *[Continue with Q4, GD]*

Over 75 years ( ) *Please end*

✓ *Women only for GD 2 and 4*

Q4. Do you or someone in your immediate circle of friends/family work in one of the following areas?

Market research, marketing, advertising, PR ( )*

In a media organisation such as radio, newspaper, TV, magazine etc. ( )*

Psychiatry, psychotherapeutic outpatient clinic, psychotherapeutic practice, counselling, etc. ( )*

None of them ( )

✓ *If one of the answers is marked with *, please finish*

✓ *Please enquire about current employment at this time.*
No unemployed

Q5. When was the last time you took part in a product survey, focus group, interview, etc.? On which topic? At which institute/studio?

Topic	Where did this take place?	Date & Time

✓ *Please only participants who have not taken part in any market research on the topic of mental health in the last 12 months.*

✓ *If the person has participated on the topic 'mental health', please finish!*

Willingness to participate in suicide prevention

Q6. TW: For the next questions, I would like to point out that the subject matter is one that can lead to a negative emotional reaction in some people. This is the topic of suicide prevention.

Read out: I know that this can sometimes be a very stressful topic, so we won't go into it in depth at this point. You can also be sure that your data will be anonymised and subject to the highest level of confidentiality, so that no personal data can be traced back to you in the course of potential participation in a scientific study.

We - Quotapoint - are recruiting on behalf of the University of Bielefeld and the market and social research institute GIM from Heidelberg. The joint study is about testing specially developed online content designed to improve suicide prevention work in the population. For you as a potential participant, this would mean that you would test the prepared information on prevention work.

Is this a topic that is okay for them if we continue the conversation now?

Yes, please continue	( ) *Continue with Q7*
No, I don't want to talk about it any further	( ) *EXIT*

Q7. Thank you for deciding to continue the conversation.
Do you feel able and are you willing to talk about suicide prevention during an online interview or online group discussion?

☞ *Ask quotas according to items, e.g. [Men]=only ask men*

[Men]: I can imagine talking about this in a confidential Individual interview-an interview-to talk	( )* *Further social input Q8*
[Men]: I can imagine in a one-to-one discussion/interview or a Group also to talk to other people about it	( ) ** Further soc.input Q8 & K.&B. to men Q10
[Men & Women]: I can imagine myself in a Group to talk about it with other people	( ) ** Continue soc.input Q8 & K.&B. to men Q10
I can't imagine talking about it	( ) *EXIT*

✓ ** Bookmark for online IDIs - 100% men*

✓ ***Note for online focus groups - 50% men, 50% women*

Social integration - only for recruitment IDIS - only men

Q8. **Next, I would like to ask you for your self-assessment. I will now read statements to you and you will use a 5-point scale to answer to what extent these statements apply to you.** 5 means 'this applies to me completely'; 1 means 'this does not apply to me at all'.

- ✓ *Please ask questions in rotation.*

Statements *[Emotional self-perception socially integrated]*	1	2	3	4	5
I can always rely on my family.					
I can always rely on my friends.					
I always get a sympathetic ear if I have any worries or problems.					
If I'm not feeling well, someone is there for me straight away.					
I don't experience the feeling of cohesion in my environment.					
Compared to others, I feel like an outsider.					

- ✓ *Good mix of participants from blue fields (low assessed social integration), yellow fields (medium assessed social integration) and red fields (high social integration)*
- ✓ *Please estimate the overall level of emotionally perceived social integration here, i.e. participants who tick 5 everywhere except for item 2 should still be counted as having a high level of social integration*

Q9. The whole thing is similar once again. I will now read statements to you and you will answer me using a scale of 5 to what extent these statements apply to you. 5 means 'this applies to me completely'; 1 means 'this does not apply to me at all'.

statements *[Factually socially integrated]*	1	2	3	4	5
I have a very large circle of friends.					
I tend to have a small circle of friends.					
I often meet up with friends.					
I don't socialise much.					
I always go out at the weekend and meet up with friends/family/colleagues					
I use my weekends for myself to regenerate. I like to keep to myself during this time.					

- ✓ *Good mix of participants from blue fields (low assessed social integration), yellow fields (medium assessed social integration) and red fields (high social integration)*
- ✓ *Please estimate de facto social integration here as a whole, i.e. participants who tick 5 everywhere except for item 2 should still be counted as having a high level of social integration*

Contact and relationship with men - only for recruitment focus groups

Q10. Next, I would like to know something about your social environment. If you think about your family, friends and acquaintances... are there any male people there with whom you have trusting relationships and regular contact, i.e. at least once a week?

Yes, there is	( ) *Continue with Q11*
No, this does not exist	( ) *Exit*

Q11. What is your relationship to this person/these people (friend, father, brother, etc.)?

Q11a: Statements *[Relationship with men]*	Please tick the appropriate box	***Q11b: I notice when something changes in his/her mood or behaviour.***	
		Applies	Does not apply
Father/Grandfather			*
Brother			*
Other male relatives			*
Friend/mate			*
Colleague (work/club)			*
Other __________			*

- ✓ *Ask for statements and have them answered, if 'applies' then add Q11b.*
- ✓ *Observe a good mix of relationships with men within the focus groups and only male persons for whom 11b also applies.*
- ✓ ** If always 'does not apply', then please end*
- ✓ *Please note quota 50% men and 50% women; in groups separately.*

Eloquence

Q12. Imagine you had the chance to meet a famous person for dinner. Who would you choose and what would you talk about?

____

____

____

____

____

- ✓ *Interviewer: Please award a mark!*
- ✓ *1,2: Participant talks spontaneously and fluently, figurative language, several sentences, imaginative*
- ✓ *3: Participant needs some time to remember/answer but is able to speak fluently and imaginatively, 2-3 sentences*
- ✓ *4: Participant can't think of anything, short description, not fluent, doesn't enjoy the question*
- ✓ *<u>Please prioritise participants with a grade of 1 or 2</u>, recruit a maximum of 25% of participants per group with a grade of 3*
- ✓ *Note 4: Please end the conversation!*

Socio-demographics

- ✓ *Please pay attention to the spread of socio-demographics per GD!*

Q13. Do you live alone or with someone?

(Still) living with parents	( )
Live alone (without partner/children/others)	( )
Live with partner and child/ren	( )
Live with partner (without child)	( )
Living with child/children (without partner)	( )
Live with other people (not family members)	( )

- ✓ *Consider a good mix, especially for IDIs*

Q14. What is your highest educational qualification?

Elementary school	( )*
Highschool	( )
Vocational school	( )
University entrance degree	( )
University, University of Applied Sciences	( )

- ✓ *Good mix, especially people with a secondary school leaving certificate*

Q15. What is your current (professional) activity?
(also [school] training/studies, etc.)

____

Full-time	( )
Part-time	( )
Housewife/husband / mother / father	( )
Jobseeker	( )
Pensioner	( )
Student at university	( )
Student at school	( )

- ✓ *Ensure a good mix; not just students or full-time employees*
- ✓ *Compare occupation with Q4 (exclusion criteria)*

Q16. Where is your current place of residence?

____

- ✓ *Query **federal state and place of residence** - Ensure a good mix for:*
  Urban-rural
  north-south / east-west
  *→ **Good distribution across federal states desired**, especially eastern Germany*

Readiness and technical requirements

T1 Do you have the possibility to use a laptop or PC at home for this project?

Yes, I can use a laptop / PC at home ( )
No, I can only use a smartphone or tablet ( ) *Exit*

T2 Does your laptop / PC have a functioning webcam and microphone?

Yes, my PC / laptop has a webcam and microphone that work ( )
No, my PC / laptop does not have a webcam ( ) *Exit*
No, my PC / laptop does not have a microphone ( ) *Exit*

T3 Have you already had experience with Zoom or similar video platforms / video services for "virtual" meetings?

Yes ( )
No ( )

- ✓ *No quota, but preferential use of participants with experience*

T4 If Yes: Setting up / using the platform / video service was for me...

Very difficult ( ) *Please finish!*
Rather difficult ( ) *If possible do not invite*
Rather light ( )
Very light ( )

T5 Do you have your own e-mail address?

Yes ( )
No ( ) *Please end call*

T6 What kind of Internet connection do you have at home?

High-speed Internet access such as cable, DSL, broadband, Wlan ( )

modem	( )	*Exit*
No internet connection at home	( )	*Exit*
Other:______________________	( )	*Exit*

We would like to invite you to participate in our study.

Participation in the study includes:

#1 Engaging with an [FGD:] online learning tool with a total length of 120 min | [IDIs] content of a website with a total engagement time of 30 min

#2 After this short period of engagement, we ask you to answer a short questionnaire about *the previously viewed material* lasting about 10 minutes. You have a total of one week to answer the questionnaire and engage with the website/e-learning tool.

#3 This is followed *(a few days later)* by a 45-minute online interview | a 90-minute group discussion

Your interview would take place at __________________ at __________.

To participate in this round, you will be asked to sign a **confidentiality agreement**

Are you able and willing to participate in this study?

Yes	( )
No	( )

Say thank you and goodbye!
