# Supplementary material for: Formative Evaluation of Suicide Prevention Websites for Men: Qualitative Study with Men at Risk of Suicide and with Potential Gatekeepers
Source: JMIR Form Res. 2025 Feb 26;9:e59829. doi: 10.2196/59829 (PMC11904374; doi:10.2196/59829)
Supplement: Multimedia Appendix 2 [file formative_v9i1e59829_app2.pdf]

is filled in by the institute

Study: ______________________

Client: ______________________

Moderator: ______________________

Contact: ______________________

End customer: ______________________

Projektnumber:______________________

Area: ______________________

Dear participant,

Thank you for taking part in our study today!

We would like to point out a few things in advance:

All **personal data** collected from you **will of course be treated as strictly confidential (General Data Protection Regulation)** and will not be passed on to third parties, which means that you will remain completely anonymous during the discussion. All personal data will remain with us.

Our client may wish to gain his own impression of the course of today's discussion/online interview, which is why we would like to record the discussion/interview and make it available to the client on request. In accordance with the professional code of conduct of German market and social research, the client undertakes to use the recording only for research purposes and only for the purpose of the study. The recording will neither be passed on to third parties (not even in the form of a copy) nor shown, any attempt at de-anonymisation will be refrained from and prevented and the recording will be deleted no later than three months after receipt.

By signing below, you agree that your personal data may be stored in accordance with the GDPR, that the video and audio recording and transmission of today's study may be **passed on to the client** under the above-mentioned conditions **and that project staff will follow the discussion.** With your signature, you further confirm that your participation is **voluntary**. You are free to **withdraw from participation** at any time **without consequences.**

If you are unable to give your consent, participation in the discussion is unfortunately not possible.

You also confirm with your signature that this study is strictly confidential and you agree not to pass on any information about the customer or the project or its contents to third parties.

Please note that the use of your mobile phone during calls is only permitted in an emergency by prior arrangement. Thank you for your understanding!

PLEASE FILL IN IN BLOCK CAPITALS!

Name, Vorname :____

Straße / Hausnummer :____

Postcode / Place of residence: ____

Telephone number: ____

Occupation: ____

Industry / Specialist: ____

Date of birth: _______________Age:_______________

When was the last time you took part in a market research interview?

Never before		Within the last 6 - 12 months	
More than 24 months ago		Within the last 3 - 6 months	
More than 12 months ago		Within the last 0 - 3 months	

______________________________ ______________________________

Date Signature
