# Supplementary material for: Formative Evaluation of Suicide Prevention Websites for Men: Qualitative Study with Men at Risk of Suicide and with Potential Gatekeepers
Source: JMIR Form Res. 2025 Feb 26;9:e59829. doi: 10.2196/59829 (PMC11904374; doi:10.2196/59829)
Supplement: Multimedia Appendix 3 [file formative_v9i1e59829_app3.pdf]

Protocols for n=24 Interviews and 4 focus groups

Interviews

17.07.2023<br>16:00-16:45<br>100, IDI 01	Male, 48 years old
**Other aspects** (technique, mood, disturbances, digressions, etc.)	• Direct contact with the topic of depression and suicidal behaviour in the social environment<br>• Affected by depression in the past<br>• great openness<br>• Very lively discussion<br>• Little to no bias regardless of the topic<br>• No technical difficulties

17.07.2023<br>17:15-18:00<br>101, IDI 02	Male, 63 years old
**Other aspects** (technique, mood, disturbances, digressions, etc.)	• Suicide advocates<br>• In a precarious economic situation (immediate threat of poverty in old age)<br>• Tends to have a negative attitude towards the explicit mission of the site<br>• little motivation to give constructive feedback on the website<br>• No technical difficulties

17.07.2023<br>18:30-19:15<br>102, IDI 03	Male, 39 years old
**Other aspects** (technique, mood, disturbances, digressions, etc.)	• Very eloquent, precise answers<br>• Due to his profession he knows very young target group affected by Corona and due to age & self-discovery problems in puberty<br>• No technical problems

17.07.2023<br>19:45-20:30 103, IDI 04	Male, 24 years old
**Other aspects** (technique, mood, disturbances, digressions, etc.)	• Contact with depression through acquaintances at younger age<br>• Precise answers<br>• Had problems at the beginning of the task to open the website during the pre-task, but was able to look at it again with pictures, so no loss of knowledge during the interview.<br>• Supporter of the websites, but would like to see more concrete action-activating content, some of the content on the site seems overloaded to him

18.07.2023<br>16:00-16:45 104, IDI 05	Male, 49 years old
**Other aspects** (technique, mood, disturbances, digressions, etc.)	• Multiple attempts to access the website, initially repeated technical problems<br>• Inconsistent response: Evaluates the website as important and indispensable, but at the same time realises that there are probably already several other websites with the same objective. AND the most relevant help (DIRECT contact or dialogue with a person who can help) is missing. The site's assistance is limited to a collection of redirects.<br>• Furthermore, unprofessional appearance due to presentation and sender. More established institutions with a greater impression of competence and authority would significantly increase the credibility and trustworthiness AND the sender must be visible much earlier and more prominently (on the first page).<br>• No technical difficulties

18.07.2023<br>17:15-18:00 105, IDI 06	Male, 67 years old
**Other aspects** (technique, mood, disturbances, digressions, etc.)	• Stagnant narrative flow when answering questions, but very fluent narrative flow when telling anecdotes from 'the past'.<br>• Even experiences with suicidal thoughts at a young age due to financial hardship→ elevates depression/financial hardship/illness to a level of factors that can cause suicide. The concept of depression/financial hardship/illness as a level of factors that can justify suicide, so that it is too one-sidedly focussed on depression in terms of content in his opinion<br>• No technical difficulties

18.07.2023<br>18:30-19:15 106, IDI 07	Male, 56 years old
**Other aspects** (technique, mood, disturbances, digressions, etc.)	• Very clearly and distinctly spoken, very reflective and pointed in statements & evaluation of the website.<br>• Even experience with depressive phases in the past with suicidal thoughts up to partial planning of suicide - but without taking concrete preparatory measures.<br>• Can identify very well with the website, would have wished for her 'back then' in his depressive episodes<br>• No technical difficulties

18.07.2023<br>19:45-20:30 107, IDI 08	Male, 66 years old
**Other aspects** (technique, mood, disturbances, digressions, etc.)	• Excessive answering behaviour and not always accurate answers to questions -> more likely to 'speak from the heart'<br>• Experiences with suicidal behaviour within the family (his daughter)<br>• Sometimes difficult to understand due to background noise

19.07.2023<br>16:00-16:45 108, IDI 09	Male, 62
**Other aspects** (technique, mood, disturbances, digressions, etc.)	• Himself affected by depression and passive suicidal thoughts (due to illness he could no longer do his job)<br>• Clearly understandable, purposefully answered, dealt very precisely with the website

19.07.2023<br>17:15-18:00 109, IDI 10	Male, 49 years old
**Other aspects** (technique, mood, disturbances, digressions, etc.)	• Crisis as a teenager→ Self-discovery as a queer person; many queer people in the circle of friends, some also currently affected by crises<br>• Understandable, no technical problems

19.07.2023<br>18:30-19:15 110, IDI 11	Male, 30 years old
**Other aspects** (technique, mood, disturbances, digressions, etc.)	• Many friends have mental health problems: anxiety disorder, depression, borderline, etc.→ very informed about help platforms, causes and correlations of mental illnesses<br>• Sometimes too much theorising, not always sticking to personal opinion<br>• 2-3 short sound failures, approximately for 30s.

19.07.2023<br>19:45-20:30<br>111, IDI 12	Male, 33 years old
**Other aspects** (technique, mood, disturbances, digressions, etc.)	• Structured answers<br>• personal connection to the topic due to management position of how prevention can be approached<br>• Family member with passive and active suicidal thoughts, for years, difficult to deal with for fear of doing the wrong thing→ rarely a topic of conversation<br>• No technical problems

20.07.2023 16:00-16:45<br>112, IDI 13	Male, 30 years old
**Other aspects** (technique, mood, disturbances, digressions, etc.)	• Having been affected by depression himself in the past, this page would have been helpful for him at that time to categorise himself, to find himself again in the problem 'to make the topic more tangible' for himself<br>• No technical difficulties, very differentiated and structured answers

20.07.2023<br>17:15-18:00<br>113, IDI 14	Male, 29 years old
**Other aspects** (technique, mood, disturbances, digressions, etc.)	• Affected by psychological stress in the past himself, talks about it with close friends, but not with work colleagues because he feels he has to be 'strong' there.

	• Differentiated view of the website, helpful self-assessment and videos of those affected are good for identification<br>• dropped out of session twice, then joined mobile and call then ran more smoothly

20.07.2023<br>18:30-19:15<br>114, IDI 15	Male, 35 years old
**Other aspects** (technique, mood, disturbances, digressions, etc.)	• Uncritical and undifferentiated opinion<br>• General approval of the stimulus at all relevant levels, strongly socially desirable response behaviour<br>• Hardly any expertise that enabled them to do more than quote generalities borrowed from media reports.

20.07.2023<br>19:34 -20:30<br>115, IDI 16	Male, 36 years old
**Other aspects** (technique, mood, disturbances, digressions, etc.)	• Very reflective and well-founded opinion<br>• Was already affected myself, had professional treatment<br>• Very open, sober and differentiated discussion<br>• Welcomes the offer, has only a few formal suggestions for improvement

20.07.2023<br>19:34 -20:30 116, IDI 17	Male, 40 years old
**Other aspects** (technique, mood, disturbances, digressions, etc.)	• Latent drowsiness<br>• Very reflective and differentiated response behaviour<br>• Not affected, but very knowledgeable<br>• Suicides among friends and acquaintances<br>• Approval of the offer, only a few formal suggestions for changes<br>• No technical difficulties

20.07.2023<br>19:34 -20:30 117, IDI 18	Male, 65 years old
**Other aspects** (technique, mood, disturbances, digressions, etc.)	• Very friendly and engaging, but uncritical and undifferentiated opinion<br>• General approval of the stimulus at all relevant levels, strongly socially desirable response behaviour<br>• Hardly any expertise, lots of unreflected platitudes,<br>• No technical difficulties

21.07.2023<br>19:45 -20:30 118, IDI 19	Male, 25 years old
**Other aspects** (technique, mood, disturbances, digressions, etc.)	• Very open and differentiated communication and judgement behaviour<br>• Not personally affected, but very knowledgeable due to close contact with those affected<br>• Cases of suicide in one's own social environment<br>• Some technical and content optimisation suggestions<br>• Basic approval of the offer<br>• No technical problems

21.07.2023 postponed to 26.07.2023 18:30 -19:15 119, IDI 20	Male, 24 years old
**Other aspects** (technique, mood, disturbances, digressions, etc.)	• Differentiated communication and judgement behaviour<br>• Not affected himself, experienced a person who committed suicide in his school days; he would suspect a depressive episode in his own mother, but both grandparents and mother are of the opinion that 'just in a bad mood' and little openness for mental illnesses<br>• One brief 'freeze' of the image, after which the video ran smoothly

24.07.2023<br>16:00 -16:45<br>120, IDI 21	Male, 54 years old
**Other aspects** (technique, mood, disturbances, digressions, etc.)	• Reflective answers, very entrepreneurial perspective<br>• Indirect contact with depression through work context and 'care' as an employer→ Managers in the company with high workload<br>• No technical difficulties

24.07.2023<br>17:15 -18:00<br>121, IDI 22	Male, 51 years old
**Other aspects** (technique, mood, disturbances, digressions, etc.)	• associates suicide very strongly with death→ Talking about deaths and accidents he has already witnessed<br>• Some minor contradictions in answers, otherwise found the 'Help' tab helpful<br>• Camera no longer worked→ Interview without picture, sound clear and understandable

24.07.2023 18:30 -19:15<br>122, IDI 23	Male, 20 years old
**Other aspects** (technique, mood, disturbances, digressions, etc.)	• Clearly differentiated opinion, especially with regard to the design and layout of the videos, myself affected by psychosomatic stress in the past and had a friend with anorexia nervosa<br>• No technical problems, camera off briefly at the beginning because TN swapped contact lenses for glasses

24.07.2023<br>19:45 -20:30<br>123, IDI 24	Male, 21 years old
**Other aspects** (technique, mood, disturbances, digressions, etc.)	• Reflective answers strongly influenced by a study he once read (content approx.: men plan suicide and then cannot be dissuaded from doing so, women attempt suicide more as a 'cry for help')→ little sense seen on the page for people with suicidal thoughts, yet good for people with depression<br>• Was mentally stressed during the Covid-19 pandemic, no depression diagnosed, also speaks of mental stress in general topics in the further course of the conversation<br>• No technical difficulties

Focus Groups

26.07.2023 16:30 -18:00 h<br>GD 4 Women 41-75 years	
**Other aspects** (technique, mood, disturbances, digressions, etc.)	• Technical problems with one woman after about 30 minutes, could be solved in the end, poor internet connection, strong dialect<br>• Lively interaction, despite sequential language problems due to online setting<br>• All reflected and differentiated opinion and openly talked about topic<br>• Mostly formal suggestions for improvement, little criticism of content, perceived as rather lengthy<br>• Many are themselves relatives of people experiencing suicidal behaviour

26.07.2023<br>19:00 -20:30<br>GD 1 Men 41-75 years	
**Other aspects** (technique, mood, disturbances, digressions, etc.)	• Partial technical problems with one men, 'brief freeze', then conversation ran smoothly<br>• Lively interaction<br>• All reflected and differentiated opinion and openly talked about topic<br>• Several suggestions for improvement<br>• Approximately half are themselves relatives of people experiencing suicidal behaviour

27.07.2023<br>16:30 -18:00<br>GD 2 Women 18-40 years	
**Other aspects** (technique, mood, disturbances, digressions, etc.)	• No technical problems<br>• Very differentiated opinion, very eloquent and reflective<br>• Very well coordinated<br>• A lot of prior knowledge through own experience, interest in the topic and job<br>• Approximately half experience indirectly/directly with mental illness & suicidal experience and behaviour

	• The content of the format was perceived as important and appropriate, but more design improvements were desired, especially in the images, which lacked authenticity

27.07.2023<br>19:00 -20:30<br>GD 3 Men 41-75 years	
**Other aspects** (technique, mood, disturbances, digressions, etc.)	• Partly technical problems with video at 1 TN, which had to be solved at the beginning<br>• 1 participant also had camera problems, so he took part without video<br>• Experience with the topic of suicide/suicidal experience and behaviour through indirect or direct contact with acquaintances<br>• chatting with some participants at the start, but less constructive input in terms of content when evaluating the website elements
