# Supplementary material for: Formative Evaluation of Suicide Prevention Websites for Men: Qualitative Study with Men at Risk of Suicide and with Potential Gatekeepers
Source: JMIR Form Res. 2025 Feb 26;9:e59829. doi: 10.2196/59829 (PMC11904374; doi:10.2196/59829)
Supplement: Multimedia Appendix 4 [file formative_v9i1e59829_app4.pdf]

GIM | RELEVANCE COUNTS.

TOPIC GUIDE

UNIVERSITY OF BIELEFELD / MEN ACCESS

SUICIDE PREVENTION

ONLINE FOCUS GROUPS

EVALUATION OF THE ONLINE OFFER: WWW. HILFE-FUER-ANGEHOERIGE.DE/

Duration: 90min

Version 2 (Final), 05.07.2023

GIM PROJECT 23-0335

AGENDA

I. WELCOME AND INTRODUCTION .... 10 MIN

Welcome to

Presentation

II. WARM-UP: TOPIC OF MENTAL HEALTH .... 10 MIN

Spontaneous associations: mental health

Everyday mental health

III. EVALUATION OF ONLINE OFFER - ATTRACTIVENESS & USABILITY .... 30 MIN

Attractiveness & orientation

General quality of online content

IV. EVALUATION ELEMENTS OF THE E-LEARNING PROGRAMME

V. APPLICABILITY & RELEVANCE IN CRISIS SITUATIONS .... 30 MIN

Scenario technique - relatives of a man in crisis - perspective taking

Recommendation - External perspective

Projective target group - change of perspective

VI. WRAP-UP AND ADVICE .... 10 MIN

TOTAL 90 MIN.

Notes for customers

This document is a qualitative guide (as opposed to a quantitative questionnaire). The questions listed are therefore neither closed questions that can be answered with a simple yes or no, nor are they absolute questions. Rather, they form "directional information" in order to be able to channel the narrative flow of the interviewee. Moreover, the questions are not read out as formulated, but are adapted flexibly to the current context.

Even if we do not give a "why" after every question, it goes without saying that every answer will be discussed to the desired level of detail if appropriate.

I. Welcome and introduction 10 MIN

Welcome to

In general:

- Express appreciation for participation
- Name the client: Bielefeld University & independent MaFo Institute
- Please switch off your mobile phone & ensure a quiet environment (no sources of interference)
- Explain micro and camera rules: Unmute, except for feedback; only use chat function when requested to avoid parallel communication threads
- Reference to recording
- Data protection and anonymisation

Rules of the game:

- No wrong answers
- Talk about feelings too
- Speak without a filter
- Be creative
- Note that the moderator sometimes deliberately intervenes to bundle narrative threads (thinking about the direction of the conversation, involving more passive people, etc.).

Presentation

First name, age, profession, marital status, hobby, living situation

☞ *Allow each participant to speak briefly*

II. Warm-up: Mental health 10 MIN

Aim: short warm-up for the participants and as a basis for the subsequent concept/design work; gentle introduction

☞ *Express appreciation for participation in preliminary task.*

Moderation: Last week you had the opportunity to engage with an online offer. Firstly, a ***short round of thumbs****, each person for themselves on 3:*

How did you **like** the **online offer**?

If you were to explain the offer to a friend.
.. What do you think would be the **overarching theme of the online offer**?
How would you describe this **in your own words**?

What is this offer about? How would you describe this online offer in one sentence?

☞ *Ask participants to define and explain terms themselves (if not understood - suggest a term similar to stress on mental equilibrium...)*

Everyday mental health

Moderation: Now to your everyday life. To what extent does the topic [XY] generally play a role in your everyday life/your circle of friends and acquaintances?

Is this a topic that is discussed with friends/family?

☞ *Keep it short, each participant gives a brief input.*

III. Evaluation of online offer - attractiveness & usability 30 MIN

Objective: Evaluation of the online offer & content in terms of attractiveness, clarity/orientation, comprehensibility, quality, evaluation of the individual content/presentation methods within the four modules/videos.

☞ *Note that in the following, some of the contents of the preliminary task are reviewed and aspects are discussed again, but new aspects can also be mentioned at the same time.*

☞ *Create a reference to the content of the preliminary task*

Moderator: We've already talked a bit about the topic [XY 'mental health']. Sometimes there are difficult or challenging situations in life, e.g. situations that seem hopeless and in which people have suicidal thoughts. The online offer is intended to help relatives of men with potentially suicidal experiences and behaviour to communicate, support and understand the topic. Within the programme, you have therefore looked at a total of 4 modules, each with a different focus.

Next, I would like to get ***your overall impression*** *of the online programme before we go into detail about the individual elements.*

Attractiveness & orientation

Spontaneous first impression

Why don't you tell us about the **situation in which** you **looked at the online offer**? Did you watch all four modules at once? Did you watch them intermittently?
How long did you have time to look at them?

In general, what was your **first impression of the online offer**?

☞ *Let participants talk freely for a short time, take notes and come back to them later.*

Attractiveness | Favour

What do you **think of** the online offer?

What do you **like**? What don't you like?

Which module did you **like best**? Which one least?

What do you think of the **basic format** (division into modules, principle of digital self-learning)?

Which elements do you like/ dislike (videos, texts, info boxes, pictures (age of the men in the pictures), expert advice, etc.)?

☞ *Only briefly explore, as elements will be discussed in more detail later*

Understanding

In one sentence: In your opinion, what is the specific **idea behind the online offer**?

What is **the aim of** the tool?

In **which situations** would you visit this online offer?

How easy do you find the tool & its contents to understand? What was easy to understand at first glance? What did you stumble across while looking at it?

Which module do you find **most understandable**? Which one is the **least understandable**?

Do you already **know** such a tool **from similar contexts** (information from health insurance companies, counselling telephone, etc.)? From **other contexts**?

Quality of online content in general

☞ *Thumb test before each sub-chapter to record everyone's individual impression first. → Always ask for 'thumbs up' as a subgroup first to avoid negative 'swirls'.*

Tonality/atmosphere

How did you like the tonality/atmosphere?

How did you perceive them? What was good/bad?

Information content

How informative do you find the online offering overall?

What information is helpful? Which is **less helpful**?

What information did you **expect** to find?

What **information was missing**?

What do you think of the **way the content is presented**? What do you like? What do you like less?

Credibility

How credible do you find the content of the online offer?

What contributes to your opinion? **Which elements/content/information make the tool credible/less credible**?

Which **aspects** do you find particularly credible?

Are there points that create **doubt**?
How could these **doubts** be **overcome**?

☞ *First let them tell you, then ask them explicitly: 'expert knowledge'; facts and figures, the perspective of those affected, graphics, pictures, etc.*

Seriousness

How serious do you find the content? What **overall impression do you get in terms of seriousness**?

What do you find **serious** about the content of the tool**? What do you find less serious**? Why?

How serious do you find the **visual design** of the offer?

Contemporary/modernity

How up-to-date do you find the tool and the chosen presentation?

How contemporary/modern is the presentation of the content?

To what extent do you **expect** a modern presentation of information in this thematic context?

Consistency

☞ *Show page briefly to indicate that a new meaning section is beginning.*

How consistent do you find the presentation of content within the online offering?

To what extent is the **presentation of the content coherent** for you?

How consistent do you find the individual elements? How consistent do you find the elements **with each other** and **in comparison with each other**?

How balanced and appropriate do you find the **division into image, text, audio and video material**?

IV. Evaluation elements of the e-learning programme

Moderation: We have already discussed the online programme in general terms. Now we want to go into a little more detail and look at the individual elements of the programme, some of which you have already mentioned. I'll share my screen with you for this.

☞ *Share screen and go through PowerPoint elements from videos individually. Discuss likes, dislikes & understanding. Red-green exercise using the PowerPoint slides*

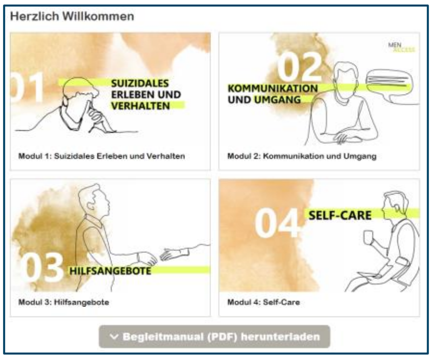

(example)

[Elements: design line art, facts & figures, graphics, e.g. relatives with pictures, experts, interviews with those affected, radio plays, pictures, take home message]

☞ *Query similar to previous question scheme*

How do you **like** XY?

What do you like about it? What **don't you like**? Why do you like it?

How comprehensible do you find this presentation?

How helpful do you find this presentation?

How much does such a **presentation affect you emotionally**? Very much, why? Not at all - what would pick you up more instead?

What do you think of the **tonality** here?

How serious do you find this presentation?

How contemporary do you find this presentation?

How informative do you find the chosen presentation?

How consistent do you find this representation in **relation to the other elements?**

What could **be improved** here**?**

V. Applicability & relevance in crisis situations 30 MIN

OBJECTIVE: Evaluation of the content with regard to applicability in crisis situations

☞ *Projective exercise - apply scenario technique and check applicability of content*

Scenario technique - relatives of a man in crisis - perspective taking

Moderator: We have already talked about the online offer in general and in detail. We will now go into a typical application situation in more detail and imagine a scenario in which an acquaintance/friend/family member/colleague of yours is in crisis.
We know that this can sometimes be a very stressful issue. So please let us know at any time if the following mental scenario is too much for you.

[Scenario 1 - FRIEND/COMPANION] You can imagine a male family member, friend, colleague or acquaintance here. Perhaps you can think of a specific example. It does not have to be the same person as the one you specified in the recruitment process. Take the person who is useful to you in the task.

This specific male person shows signs of depression. He is depressed and has little joy in everyday life, you may even notice that he has problems with alcohol consumption or everyday activities. He may even be talking about suicidal thoughts. Suppose you want to help the friend/acquaintance/etc...

To what extent would you **use** these **modules/this online offer**? **How** would you **use** the online programme **in your everyday life**?

How would this offer **help** you?

What motivates you to **try out** this online offer**?**

What would you **expect from** using such an **online service** beforehand?

To what extent does this correspond to your **current experience**?

What **helps** you in the **current version**? What is missing?

What do you **need** for **the offer** to **help** you **(even more)**?

Recommendation - External perspective

[Scenario 2 - Recommendation to acquaintances] Intervier:in: Now let's think a little further...

To what extent would you **recommend** the online offer to relatives in such situations? **When would you recommend it**? When would you not?

What is the main motivation to **recommend/not recommend** the offer?

What speaks in favour of recommending the offer? What **speaks against it**?

Which of the modules would you **recommend** the most and which the least? Why?

Which of the elements would you **recommend** the most and which the least? Why?

How action-activating do you find the **suggestions**? Do you need more tips? Should the tips **be more present at** the beginning?

In which contexts could this online offer be helpful? (e.g. as information on health insurance websites, e.g. notice in the AOK booklet, in doctors' surgeries, in clubs as a poster, etc.)

Which channels should be **used to raise awareness**?

Projective target group - change of perspective

[Scenario 3 - Target group] Moderation: Independent of your friend/acquaintance/XY...

For whom could this e-learning programme be **helpful**? Who would you **recommend** this online programme **to**? Which person would you rather not? (e.g. older, younger, severity of the crisis, etc.)

Who could use this offer?

To what extent would you **recommend** this offer to the person? How would you not?

To what extent can the **tips and suggestions** be **implemented in practice**?

How action-activating do you find the **suggestions**? Do you need more tips? Should the tips **be more present at** the beginning?

In which contexts could this offer be helpful? (e.g. as information on health insurance websites, e.g. notice in the AOK booklet, in doctors' surgeries, in clubs as a poster, etc.)

How should you find **out about this online offer**?

How would you recommend the online offer to others? (View together, browse alone, etc.) How would you **not recommend** the online offer?

To what extent do you have **reservations about recommending** this online offer **to others**? What speaks in favour? What speaks against it?

VI. Wrap-up and advice 10 MIN

Moderation: We are now almost at the end of the discussion...

To summarise again: **What** do you think needs to be **improved about this online offering** and its content?

What would you **like to say to the clients of** the study **in conclusion**?

In your opinion, are there any **topics/aspects that** were **not sufficiently highlighted in the discussion**?

Moderation: In addition to this online offer of help for men in crisis situations, there are also other similar offers.

Are you **aware of any other such offers of help**? If yes, which ones? If no, please name them directly.

Finally, I would like to point out that in addition to this information in the context of suicide prevention, there are also other options, such as the possibility of getting help in crisis situations, other offers of help, e.g. the telephone counselling service on *0800-1110 222/111, the online telephone counselling service in Germany or the websites of larger health insurance companies, such as the AOK or Techniker.*

Say thank you and goodbye!
