# Supplementary material for: Formative Evaluation of Suicide Prevention Websites for Men: Qualitative Study with Men at Risk of Suicide and with Potential Gatekeepers
Source: JMIR Form Res. 2025 Feb 26;9:e59829. doi: 10.2196/59829 (PMC11904374; doi:10.2196/59829)
Supplement: Multimedia Appendix 5 [file formative_v9i1e59829_app5.pdf]

GIM | RELEVANCE COUNTS.

TOPIC GUIDE

UNIVERSITY OF BIELEFELD / MEN ACCESS

SUICIDE PREVENTION

ONLINE INTERVIEWS

EVALUATION OF THE WEBSEITE MAENNER-STAERKEN.DE

Duration: 45min

Version 3 (FINAL), 05/07/2023

GIM PROJECT 23-0335

AGENDA

I. WELCOME AND INTRODUCTION .... 5 MIN

Welcome to

Presentation

II. WARM-UP: TOPIC OF MENTAL HEALTH .... 5 MIN

Spontaneous associations: mental health

Everyday mental health

III. WEBSITE TEST - DESIGN, INTUITIVENESS, CONTENT .... 18 MIN

Attractiveness & orientation

Quality of content

IV. APPLICABILITY & RELEVANCE IN CRISIS SITUATIONS .... 15 MIN

Scenario technique - friend in crisis - distant perspective

Projective target group - change of perspective

Own crisis - adopting a perspective

V. WRAP-UP AND ADVICE .... 2 MIN

TOTAL 45 MIN.

Notes for customers

This document is a qualitative guide (as opposed to a quantitative questionnaire). The questions listed are therefore neither closed questions that can be answered with a simple yes or no, nor are they absolute questions. Rather, they form "directional information" in order to be able to channel the narrative flow of the interviewee. Furthermore, the questions are not read out as formulated, but are adapted flexibly to the current context.

Even if we do not give a "why" after every question, it goes without saying that every answer will be discussed to the desired level of detail if appropriate.

I. Welcome and introduction 5 MIN

Welcome to

In general:

- Express appreciation for participation
- Name the client: Bielefeld University & independent MaFo Institute
- Please switch off your mobile phone & create a quiet, undisturbed environment
- Reference to recording
- Note on camera function: leave camera on (offer if you prefer without camera, also fine - both IDI forms are possible) in order to be able to capture several levels of communication on both sides (serves the atmosphere of the conversation) - later, transcripts are used so that the face is not recognisable.
- Data protection and anonymisation

Rules of the game:

- No wrong answers
- Talk about feelings too
- Speak without a filter
- Be creative

Presentation

First name, age, profession, marital status, hobby, living situation

II. Warm-up: topic of mental health 5 MIN

Aim: short warm-up for the participants and as a basis for the subsequent concept/design work; gentle introduction

Moderation: According to studies, almost 30% of the adult population in Germany is affected by a mental illness every year. This is often caused by lack of time, stress, alienation and fear of the future. These numerous causes can unbalance the soul.
We are dealing here with a phenomenon that affects the entire population and, in the worst case, can even lead to suicide
if the pressure and stress become
too great. Current studies show that men in particular are at risk of suicide.
Although society's understanding of mental stress is increasing nowadays, there is still a long way to go in terms of adequate and comprehensive treatment options. This is why our study aims to make a positive contribution by talking to people in the population about this topic.

We know it is difficult to name this topic...

What is **the term/name of the topic you have just addressed** that you can best work with in the following conversation?

☞ *Let the participants define their own terminology (if not understood - suggest a term similar to stress on mental equilibrium...)*

Spontaneous associations: mental health

Spontaneous associations with the keyword '[XY - named by participant]'?
What images come to mind?
What is connected with it?

Everyday mental health

To what extent does the topic '[XY]' generally play a role in your everyday life?

Is this a topic that is discussed with friends/family?

☞ *Keep short*

III. Website test - design, handling & content 18 MIN

Objective: Evaluation of website elements & content in terms of attractiveness, clarity/orientation, comprehensibility, intuitiveness & quality

☞ *Firstly, express appreciation for participation in the preliminary task & point out that some of the content will be gone through again below and aspects discussed again, but that new aspects can also be mentioned at the same time.*

☞ *Create a reference to the content of the preliminary task*

Moderator: We've already talked a bit about the topic [XY - mental health]. Sometimes there are difficult or challenging situations in life, for example situations that seem hopeless and in which people have suicidal thoughts. The website that was developed in the project is intended to help men in such crisis situations .

Attractiveness & orientation

Spontaneous first impression

Why don't you tell us about the **situation in which** you were **involved with the website**? How long did you have to work on it?

In general, what was your **first impression of the website**?

☞ *Let participants talk freely for a short time, take notes and come back to them later.*

Attractiveness | Favour

How do you **find** the site?

What do you **like**? What don't you like?

Which elements do you like/ dislike (videos, texts, info boxes, pictures (age of the men in the pictures), expert advice, etc.)?

Understanding

In one sentence: In your opinion, what is the specific **idea behind the website**?

What is the aim of the website?

In **which situations** would you visit this website?

How easy did you find the site & its content to understand? What was easy to understand at first glance? What did you stumble across while looking at it?

Which elements did you **find** particularly **understandable**, and why? Which ones less so, why?

What should have been changed to make the relevant elements easier to understand? **What** can be **improved**?

Orientation/clarity & intuitive accessibility

☞ *Briefly display the website and talk through what was noticed first, what was searched for and make a note of it*

Did you **find** everything **at first glance**? If yes, what were you looking for?
If not, how did you proceed?

How intuitive do you find the website?

What was missing?
What do you miss on the website?

To what extent were there **moments** for you **when you would not have continued looking**? **Were there moments when you had to quit**? If yes, where? If not, why not?

How did you **find** your way around the site? **How clear** do you find the structure of the site?

Which content and **design elements contribute** to easy orientation? Which do not?

What do you think of the **menu navigation**?

How do you find the **structure of the website** (large image banner, videos, text box underneath, etc.)?

How do you think the site could be **organised more clearly**?

Quality of content

Tonality/response

How did you like the address on the website?

How did you perceive them? What was good/bad?

☞ *First let them tell their story, then explicitly ask for 'you' and general factual mention and third person (about men)*

How do you like the change in address? (direct/3rd person over men, factual mentions, etc.)

Information content

How informative do you find the website?

What information is helpful? Which is **less helpful**?

What information do you **expect to** find on this website?

What **information was missing**?

What do you think of the **way the content is presented**? What do you like? What do you like less?

Credibility

How credible do you find the website?

What contributes to your opinion? **Which elements/content/information make the website credible/less credible**?

Which **aspects** do you find particularly credible?

Are there points that create **doubt**?
How could these **doubts** be **overcome**?

☞ *First let them tell you, then ask explicitly: 'expert knowledge'; statistics, perspective of those affected through videos*

Seriousness

How reputable do you find the website and its content? What is **your overall impression** of the **seriousness of** the website?

What do you find **serious** about the content of the website? **What do you find less serious**? Why?

How serious do you find the **visual design** of the website?

How does the development of the website (by men for men) affect you?

Do you know the institutions behind MEN ACCESS? What do you think of them?

Contemporary/modernity

How up-to-date do you find the website?

How contemporary/modern is the presentation of the content?

To what extent do you **expect** a modern presentation of information in this thematic context?

Consistency

☞ *Show page using specific examples and elements of the website: menu navigation, image banner home/overview; image, text and video layout*

How consistent do you find the presentation of content on the website?

To what extent is the **presentation of the content coherent** for you?

How consistent do you find the individual elements of the website? How consistent do you find the elements **among each other** and **in comparison with each other**?

How appropriate do you find the **images**?

How coherent do you find the **subdivision in the menu navigation**?

How balanced and appropriate do you find the **division into image, text and video material**?

IV. Applicability & relevance in crisis situations 15 MIN

OBJECTIVE: Evaluation of the content with regard to applicability in crisis situations

☞ *Projective exercise - apply scenario technique and check applicability of content*

Scenario technique - friend in crisis - distant perspective

Interviewer: We've already talked about the website in general. We will now go into a typical application situation in more detail and imagine a scenario in which a friend of yours is in crisis.
We know that this can sometimes be a very stressful issue. So please let us know at any time if the following scenario is too much for you.

[Scenario 1 - FRIEND/COMPANION] You can imagine a male family member, friend, colleague or acquaintance here. Perhaps you can think of a specific example. It does not have to be the same person as the one you specified in the recruitment process. Take the person who is useful to you in the task.

This specific male person shows signs of depression. He is depressed and has little joy in everyday life, you may even notice that he has problems with alcohol consumption or everyday activities. He may even be talking about suicidal thoughts.

To what extent would you **recommend** this **website to your friend** (acquaintance/XY)? How would you not?

How would you recommend the website to others? (View together, browse alone, etc.) How would you **not recommend** the website?

What would **motivate** you **to recommend this website to others**?

Where do you think the **website** should **be located/recommended**?

Which elements/aspects of the website would you **particularly recommend** to your friend (the acquaintance/XY)? Why? Which not?

Projective target group - change of perspective

[Scenario 2 - Target group] Intervier:in: Independent of your friend/acquaintance/XY...

For whom could this website be **helpful**? Who would you **recommend** this website **to**? Which person would you rather not? (e.g. older, younger, severity of the crisis, etc.)

Who could use this website?

To what extent would you **recommend** this **website to your friend** (acquaintance/XY)? How would you not?

To what extent can the **tips and suggestions on the website** be put **into practice?**

How action-activating do you find the **suggestions**? Do you need more tips? Should the tips **be more present at** the beginning?

In which contexts could this website be helpful? (e.g. as information on health insurance websites, e.g. notice in the AOK booklet, in doctors' surgeries, in clubs as a poster, etc.)

How should you find **out about this website**?

How would you recommend the website to others? (View together, browse alone, etc.)
How would you **not recommend** the website?

To what extent do you have **reservations about recommending** this **website to others**? What speaks in favour? What speaks against it?

Own crisis - adopting a perspective

[Interviewer:in And now, purely hypothetically, what would it be like for you if you yourself [were affected by mental health problems/ XY...]?

To what extent would you find the **website helpful if you** were **in a crisis situation yourself**? What information would you find helpful? Which less so?

To what extent do you feel **emotionally engaged by the website**? Rather more/less? Why?

Which **elements** of the website do you find **helpful**? Which ones less so?

To what extent do you feel **that** the **advice and tips on the website can be realistically implemented**?
To what extent **are they suitable for everyday use**?

To what extent would this be a **relevant source of information for** you in the event of a crisis? In what way would it not?

Which of the **information was already known to you**? Which was new to you?

How would you like to have found this website?

(If not already done and only if there is still time)
Would you like to tell us briefly **about your personal experiences with life crises and to what extent such a website could have helped you** (or not)?

V. Wrap-up and advice 2 MIN

Interviewer:in: We are now almost at the end of the interview...

Summarised once again: **What** do you think needs to be **improved on the website** and its content?

What would you **like to say to the clients of** the study **in conclusion**?

In your opinion, are there any **topics/aspects that** were **not sufficiently highlighted in the interview**?

Interviewer:in: In addition to this website as an offer of help for men in crisis situations, there are also other similar offers. Are you **aware of any other such offers of help**? If yes, which ones? If no, please name them directly.

Finally, I would like to point out that, in addition to this website, there are other ways to get help in crisis situations, such as the telephone counselling service on 0800-1110 222/111, the online telephone counselling service in Germany or on the websites of larger health insurance companies such as AOK or Techniker.

Say thank you and goodbye!
